# Ultrasonographic Measurement of Mean Cross-sectional Area of the Median Nerve in Pregnant Women in a Tertiary Level Hospital of Nepal: A Descriptive Cross-sectional Study

**DOI:** 10.31729/jnma.6134

**Published:** 2021-02-28

**Authors:** Govinda Adhikari, Prakash Kayastha, Sundar Suwal, Sharma Paudel, Dinesh Chataut, Santosh Maharjan, Ghanshyam Gurung

**Affiliations:** 1 Department of Radiology, Grande International Hospital, Dhapasi, Kathmandu, Nepal; 2 Department of Radiology and Imaging, Tribhuvan University Teaching Hospital, Maharajgunj, Kathmandu, Nepal

**Keywords:** *carpal tunnel*, *cross sectional area*, *median nerve*, *ultrasound*

## Abstract

**Introduction:**

Pregnancy is one of the predisposing factors for carpal tunnel syndrome, which may manifest as swelling of the median nerve. The purpose of this study was to obtain the mean cross-sectional area of median nerve using ultrasound in pregnant females at carpal tunnel inlet and its variations with different trimesters.

**Methods:**

A total of 102 pregnant females at first, second and third trimesters were evaluated for median nerve with ultrasonography. Mean cross sectional area of median nerve was calculated in both hands by using two methods-direct and indirect. The study was conducted after obtaining ethical clearance from Institutional Review Board of Institute of Medicine, and after obtaining the written informed consent from the subjects. Convenience sampling technique was used. Data obtained were compiled and analyzed using SPSS.

**Results:**

The overall mean cross-sectional area was 6.84±1.09 mm^2^ using direct method and 7.09±1.19 mm^2^ using indirect method. The values obtained with indirect method were greater than that obtained with direct method. Mean cross-sectional area of the median nerve were higher at third trimester in both hands and by both direct and indirect methods.

**Conclusions:**

From the study conducted, the normal value of mean cross-sectional area of median nerve in pregnant females was established.

## INTRODUCTION

Median nerve is subjected to compression in the carpal tunnel giving rise to constellation of symptoms known as carpal tunnel syndrome (CTS). CTS is most prevalent in the middle age females, one of the predisposing factor being pregnancy.^1^

Various parameters have been described regarding the use of US for the diagnosis of CTS of which the cross sectional area (CSA) at the entrance of the carpal tunnel seems to have the highest diagnostic sensitivity and specificity.^2^

Studies have shown that median nerve impairment occurs even in asymptomatic pregnant females using nerve conduction studies.^3^ The CSA of the median nerve in pregnant females without symptoms and signs of CTS has not been evaluated till date to our knowledge. The purpose of this study is to measure the mean CSA of the median nerve at the carpal tunnel inlet in asymptomatic pregnant females of different gestational ages.

## METHODS

This was a descriptive cross-sectional study conducted in a total of 102 pregnant females of different gestational age group categorized into first, second and third trimesters with 34 females in each trimester.

Pregnant females were referred for the obstetric ultrasound in different trimesters as a part of routine obstetric examination. The study was conducted after obtaining ethical clearance from Institutional Review Board of Institute of Medicine, and after obtaining the written informed consent from the subjects. The study was conducted in Department of Radiology and Imaging of Tribhuvan University Teaching Hospital during the period of October 2014 to September 2015. Convenience non-probability sampling technique was used. Subjects with any pathology in wrist and clinical suspicion of carpal tunnel syndrome were excluded from the study. The cross-sectional area of the median nerve in both wrists at the carpal tunnel inlet was measured by using high frequency linear array transducer (L17-5) of Phillips-iU-22 ultrasound machine.

For the measurement of the median nerve at wrist, axial images were obtained at the level of the pisiform bone, and the image with the optimal definition of the borders of the median nerve was selected. Median nerve CSA measurements were performed from the inner border of the perineural echogenic rim using two measuring methods: 1. The indirect method (ellipsoid formula) calculating the transverse and anteroposterior diameters; and 2. The direct method (tracing method). Sample size calculation was done using the formula


$$\begin{array}{ll} \text{n} & ={\text{Z}}^{\text{2}}\times \text{p}\times \text{q}/{\text{e}}^{\text{2}}\\ & =1.96^{2}\times 0.5\times 0.5/0.1^{\text{2}}\\ & =96 \end{array}$$

Where,

n = sample size
Z = 1.96 at 95% Confidence Interval
p = prevalence of elderly malnutrition i.e. 24%^16^
q = 1-p
e = margin of error 10%

Data obtained were compiled and analyzed using standard statistical analysis. Microsoft Excel and SPSS 20 was utilized for the data analysis and presentation. Mean CSA of the median nerve of each hand were calculated in pregnant females at first, second and third trimester. Overall mean CSA for all the groups was calculated by adding the values from right and left hand.

## RESULTS

Among the 102 pregnant females, 34 each were in first, second and third trimester. Maximum number of the subjects were of age group 20-24 years, 43 (42.1%) and minimum of age group 35-40 years, 3 (2.9%). Minimum age of pregnant female was 20 years and maximum age was 36 years with mean age of 25.97±4.18 years.

The mean CSA by direct method was 6.84±1.09 mm^2^ and using indirect method was 7.09±1.19 mm^2^ (Table 1).

**Table 1:** Overall mean and range of CSA of the median nerve using direct and indirect methods (n=102).

Method	Mean±SD (mm^2^)	Maximum (mm^2^)	Minimum (mm^2^)
Direct Right hand	6.84±1.09	11.2	4.7
Direct Left hand	6.83±1.19	11.7	4.4
Direct Total	6.84±1.09	11.7	4.4
Indirect Right hand	7.20±1.36	12	4.7
Indirect Left hand	6.98±1.34	11.7	4.4
Indirect Total	7.09±1.19	11.7	4.4

The mean CSA was 6.84±1.19 mm^2^ and 6.83±1.19 mm^2^ in right and left hands respectively using direct method. Using indirect method, the values were 7.20±1.36 mm^2^ and 6.98±1.34 mm^2^ in right and left hands respectively (Table 1).

The mean CSA of the median nerve by using direct and indirect methods in both hands at different levels of pregnancy are tabulated (Table 2).

**Table 2:** Mean CSA of the median nerve at various periods of gestation (n = 102).

Mean CSA (mm^2^)			
	First trimester	Second trimester	Third trimester
Direct Method Right	6.67±1.24	6.82±1.29	7.04±1.04
Indirect Method Right	6.91±1.20	7.29±1.51	7.42±1.32
Direct Method Left	6.50±0.88	6.88±1.32	7.12±1.27
Indirect Method Left	6.52±1.08	7.22±1.53	7.20±1.28

Mean CSA of the median nerve were higher at third trimester in both hands and by both direct and indirect methods.

## DISCUSSION

The diagnosis of carpal tunnel syndrome is usually done on the basis of clinical symptoms and various physical examinations and is confirmed by nerve conduction study which is the most reliable diagnostic tool.^4^ Ultrasound (US) criteria for median nerve compression include the classic triad of nerve flattening in the distal tunnel, nerve swelling at the distal radius or (less frequently) in the proximal tunnel, and palmar bowing of the flexor retinaculum.^5^ However cross sectional area (CSA) at the entrance of the carpal tunnel seems to have the highest diagnostic sensitivity and specificity.^2^ A good correlation has been demonstrated between the area of the median nerve measured with US and the severity of electromyographic findings or the functional outcome after surgery.^6^

We measured cross sectional area of the median nerve in pregnant females, one of the predisposing factors for carpal tunnel syndrome, using two methods-direct and indirect. The overall mean CSA of the median nerve using direct method was 6.84±1.09 mm^2^ and using indirect method was 7.09±1.19 mm^2^ with a range of 4.65-11.4 mm^2^ by direct method and 4.55-11.35 mm^2^ by indirect method. Wanitwattanarumlug B et al^7^ found similar results (mean CSA 6.83 +/- 0.98 mm^2^ by direct method and 6.81 +/- 1.12 mm^2^ by indirect method) as our study. However, Mani B et al^8^ found slightly higher mean CSA (7.4 mm^2^) than mean CSA in our study. Mean CSA in our study is lower than the cutoff derived by Moran et al^9^ for exclusion of CTS (11 mm^2^ and 9.8 mm^2^ for direct and indirect methods respectively). None of our subjects had CSA equal to or greater than 13 mm^2^ (direct) and 12.3 mm^2^ (indirect), which was diagnostic of CTS in their study.

The incidence of pregnancy related CTS is variable and ranges from 0.34% (Stolp-Smith et al)^10^ to 62% (Padua et al)^11^ depending on the study design and the criteria for diagnosis. Up to 62% of pregnant women report hand symptoms in third trimester.^11^ We also found slight higher mean CSA of median nerve of the pregnant females at third trimester than first and second trimesters.

Mean CSA of the median nerve in either hand using both direct and indirect method were similar in our study. This finding is in agreement with the study done by Wanitwattanarumlug et al^7^ where they found no significant difference in the mean CSA between right and left hand. The value of mean CSA obtained by indirect method in present study was higher than that obtained by direct method in both right and left hand. This finding is in contradiction with the studies performed by Wanitwattanarumlug et al^7^ and Aleman et al^12^ where they concluded no significant difference in the mean CSA by either methods. Also Moran et al9 and Yesildag et al^13^ reported that the measurements obtained with the direct method show higher values than those obtained with the indirect method, which was in contradiction to our finding as we found higher values with indirect method. Wong et al^2^ stated that sonography is an operator-dependent test, and appropriate experience is required for it to be reliable and reproducible. The differences in the value obtained may be due to the examination techniques, the transducers used or the expertise in the corresponding field. As median nerve is not perfectly ellipsoid in shape and we took the largest anteroposterior and transverse diameter for the calculation of CSA by indirect method, this may be the cause for higher values obtained in present study. Moreover a number of studies have reported that the direct method has greater diagnostic reliability than the indirect method (ellipsoid formula).^1,14^ Among the key aspects of the sonographic examination protocol are definition of the margins of the median nerve and selection of an image that permits this delimitation.^12^

## CONCLUSIONS

Sonographic examination of the median nerve and measurement of its CSA is a useful diagnostic tool in the evaluation of CTS. US has advantage of easy availability, low cost, quick scan time, able to scan a long segment of nerve and examine the structures in both static and dynamic state. Normal mean CSA of asymptomatic non pregnant females has been established by present study, which also showed higher mean CSA of median nerve at third trimester.
